# Environmental Tobacco Smoke: The Colic Connection

**Published:** 2005-02

**Authors:** M. Nathaniel Mead

The ancient Greeks were the first to describe infant colic, a condition of inconsolable crying that may afflict as many as 28% of U.S. infants, yet the root causes of this malady remain a mystery. Among the theoretical risks are milk and soy allergies, infant temperament, and problems with the infant–caregiv-er interaction. Researchers now speculate that maternal smoking during or after pregnancy may result in gastrointestinal (GI) dysregula-tion and thus increase the risk of infant colic.

This hypothesis, proposed by Brown University epidemiologist Edmond Shenassa and Mary Jean Brown, an adjunct assistant professor of society, human development, and health at Harvard, is based on a review of more than 80 papers in different fields. As reported in the October 2004 issue of *Pediatrics*, the focus of the researchers’ attention was the gut protein motilin. Infants with colic have higher motilin levels independent of how they are fed, suggesting this protein may play an important physiologic role in colic and other GI-related disorders among babies.

Three distinct lines of evidence were used to construct the hypothesis. First, mothers who smoke are twice as likely to have infants with colic. Second, exposure to nicotine and other tobacco smoke metabolites causes higher-than-average blood motilin concentrations. Finally, pediatric studies suggest that high motilin levels may predispose infants to colic.

Each year, more than 500,000 U.S. infants are born having already been exposed to secondhand smoke metabolites *in utero*. Many more are exposed in the first few years after birth. Exposure through breast milk may be just as serious as exposure *in utero*, because nicotine concentrations in breast milk may be up to three times higher than maternal blood levels.

Further, says Brown, “Even if mothers don’t smoke, the impact of [their own exposure to] secondhand smoke may be substantial.” However, she says, there are no studies in which maternal exposure to environmental tobacco smoke has been directly quantified and linked to risk for colic.

It also remains to be seen whether smoking interacts with other GI reactivity factors, such as milk allergy. “Given the available data, we can’t really speculate about possible interactions,” says Brown, “nor do we know what underlying mechanisms might cause interactions between risk factors.”

Shenassa is presently conducting a prospective population-based study, recruiting pregnant mothers and following them for about eight weeks after birth. This will be the first study to carefully consider the effects of both smoking and motilin on infant colic.

“It is significant that colic might have an environmental basis,” says S. Katharine Hammond, a professor of environmental health sciences at the University of California, Berkeley. “Unlike the costs of controlling many environmental hazards, *in utero* exposures to environmental tobacco smoke can be minimized with little cost through education and restricting smoking in workplaces and public places.”

## Figures and Tables

**Figure:**
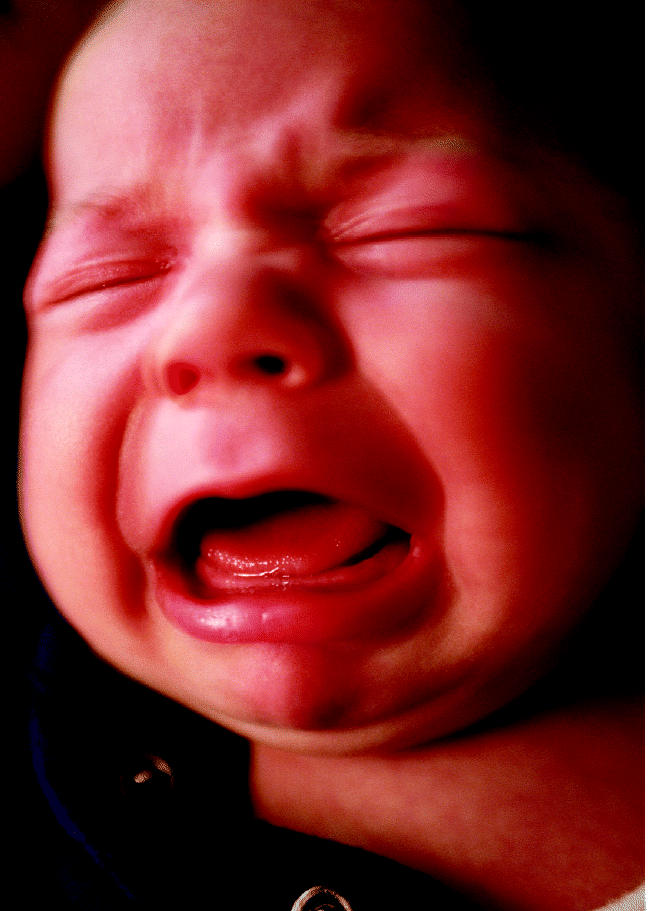
**A factor in fussiness.** A literature review suggests that maternal smoking may lead to colic.

